# Valproic Acid as a Potential Inhibitor of *Plasmodium falciparum* Histone Deacetylase 1 (*Pf*HDAC1): An *in Silico* Approach

**DOI:** 10.3390/ijms16023915

**Published:** 2015-02-11

**Authors:** Mohamed A. Abdallah Elbadawi, Mohamed Khalid Alhaj Awadalla, Muzamil Mahdi Abdel Hamid, Magdi Awadalla Mohamed, Talal Ahmed Awad

**Affiliations:** 1 Department of Pharmacology, Faculty of Pharmacy, University of Khartoum, Khartoum 11111, Sudan; E-Mail: mabadawi@uofk.edu; 2 College of Pharmacy, University of Hail, Hail 81451, Saudi Arabia; 3 Department of Parasitology and Medical Entomology, Institute of Endemic Diseases, University of Khartoum, Khartoum 11111, Sudan; E-Mail: mahdi@iend.org; 4 Department of Pharmaceutical Chemistry, Faculty of Pharmacy, University of Khartoum, Khartoum 11111, Sudan; E-Mail: mawadalla@uofk.edu; 5 Medicinal and Aromatic Plants Research Institute, National Centre of Research, Khartoum 11111 Sudan; E-Mail: talaladlan@hotmail.com

**Keywords:** *Pf*HDAC1, malaria, valproic acid, histone deacetylase inhibitor, homology model, docking, molecular dynamics, binding energy

## Abstract

A new *Plasmodium falciparum* histone deacetylase1 (*Pf*HDAC1) homology model was built based on the highest sequence identity available template human histone deacetylase 2 structure. The generated model was carefully evaluated for stereochemical accuracy, folding correctness and overall structure quality. All evaluations were acceptable and consistent. Docking a group of hydroxamic acid histone deacetylase inhibitors and valproic acid has shown binding poses that agree well with inhibitor-bound histone deacetylase-solved structural interactions. Docking affinity *dG* scores were in agreement with available experimental binding affinities. Further, enzyme-ligand complex stability and reliability were investigated by running 5-nanosecond molecular dynamics simulations. Thorough analysis of the simulation trajectories has shown that enzyme-ligand complexes were stable during the simulation period. Interestingly, the calculated theoretical binding energies of the docked hydroxamic acid inhibitors have shown that the model can discriminate between strong and weaker inhibitors and agrees well with the experimental affinities reported in the literature. The model and the docking methodology can be used in screening virtual libraries for *Pf*HDAC1 inhibitors, since the docking scores have ranked ligands in accordance with experimental binding affinities. Valproic acid calculated theoretical binding energy suggests that it may inhibit *Pf*HDAC1.

## 1. Introduction

Malaria, the life-threatening parasitic disease, is responsible for 627,000 deaths worldwide annually [1]. In humans, the disease is caused by different *Plasmodium* sp., namely *P. falciparum*, *P. vivax*,* P. ovale*,* P. malariae* and* P. knowlesi*, with* P. falciparum* being the major cause of malaria deaths worldwide [1]. Currently, the World Health Organization (WHO) recommends artemisinin-based combination therapies (ACTs) as the first line treatment for severe malaria. Nevertheless, the emergence of resistance to ACTs has called for the search for new antimalarials [2].

In eukaryotes, histone deacetylases (HDACs) are part of the epigenetic machinery, which controls important biological processes, like proliferation and differentiation, through the control of gene expression. HDACs regulate chromatin remodeling by removing the acetyl group from the ε-amino side chain of several lysine residues of the histone protein, allowing the DNA wrapped around histones to unfold and be accessible for transcription factors. HDACs also regulate gene expression together with some acetylases by deacetylation/acetylation of other non-histone proteins, such as transcription factors [3]. In humans, the HDAC superfamily is classified into four groups based on function and sequence similarity to yeast prototypes: HDAC1, HDAC2, HDAC3 and HDAC8 constitute class I; HDAC4, HDAC5, HDAC6, HDAC7, HDAC9 and HDAC10 belong to class II; HDAC11 is the sole member of class IV; these three groups are related to the zinc-dependent yeast Rpd3 or Hdac1, whereas class III is related to the NAD^+^-dependent yeast silent information regulator protein 2 (Sir2), also called sirtuins, and includes Sirt1–Sirt7 [4,5]. In *Plasmodium falciparum*, two HDAC proteins were characterized, *Plasmodium falciparum* histone deacetylase 1 (*Pf*HDAC1) and *Plasmodium falciparum* sirtuin 2 (*Pf*Sir2), which are homologues to class I and class III, respectively, but none of their structures have been solved [6,7].

Because of their critical role in the regulation of essential biological processes, HDACs are well recognized as a cancer therapy target. The hydroxamic acid-based HDAC inhibitor, suberoylanilide hydroxamic acid (SAHA), is approved in the treatment of cutaneous T-cell lymphoma [8]. There is a promising body of experimental data investigating the effect of HDAC inhibitors, particularly hydroxamic acid derivatives, against several parasites, including *Plasmodium falciparum*, where the HDACs were validated as a therapeutic target, and *Pf*HDAC1 is likely the target of hydroxamate inhibitors [9,10,11,12,13].

The old anticonvulsant and mood stabilizer, valproic acid, has been found to inhibit zinc-dependent class I human HDACs [14]. Interestingly, valproic acid was also found to inhibit the *in vitro* growth of *Toxoplasma gondii* and was proven to have HDAC-mediated activity against miracidia of *Schistosoma mansoni* [15,16]. No published experimental data are available for valproic acid inhibition of *Pf*HDAC1, except an unpublished IC_50_ of 100 µM reviewed by Andrews *et al*. [9]. In mammalian cells, the reported IC_50_ of HDAC inhibition by valproic acid was 433–1350 µM compared to 5–20 µM of the licensed drug, SAHA [9]. Considering the promising results currently obtained in clinical trials investigating valproic acid as a potential therapy for different cancers together with the valproic acid maximum dose that can reach 60 mg/kg/day [17,18,19,20], we hypothesize that valproic acid may have an activity against *Pf*HDAC1.

**Figure 1:**
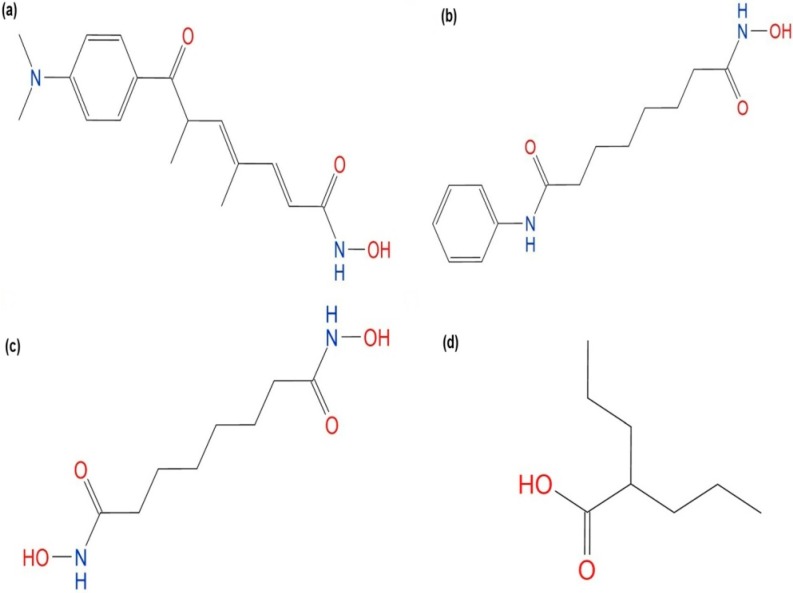
Two-dimensional structures of the ligands used in docking work. (**a**) Trichostatin A (TSA); (**b**) suberoylanilide hydroxamic acid (SAHA); (**c**) suberoyl bis-hydroxamic acid (SBHA); and (**d**) valproic acid.

In this work, a *Pf*HDAC1 homology model was built, and the model quality was assessed. The model active site architecture has been investigated and evaluated by docking of known hydroxamate *Pf*HDAC1 inhibitors reported in the literature [10]; as seen in Figure 1. Further, valproic acid was docked; the generated docking poses were compared; and the theoretical binding energies were calculated and compared to available experimental data.

## 2. Results and Discussion

### 2.1. Model Building and Refinement

To find a template protein structure for building the *Pf*HDAC1 homology model, the *Pf*HDAC1 sequence obtained from UniprotKB (Accession Number Q7K6A1) was used to query the sequences of structures deposited in the Protein Data Bank (PDB) using the protein Basic Local Alignment Search Tool (BLAST) [21,22,23]. Human HDAC2 structure (PDB:3MAX) was found to have the highest sequence identity (63%) with *Pf*HDAC1 [24]. Previously, three different *Pf*HDAC1 models were generated using two templates for each: The first was derived from (PDB: 3MAX) and human HDAC8 (PDB: 1T69); the other two models were constructed from the former template and the yeast HDAC-like protein (1C3R) [10,11,25]. The latter two templates share 41% and 31% sequence identity with *Pf*HDAC1, respectively. A multiple sequence alignment of the target and the described templates using ClustalX [26] is shown in the Figure S1, where (PDB: 3MAX) clearly has the highest sequence identity to and coverage of *Pf*HDAC1.

The final intention for building a homology model is to predict an unknown protein structure from its sequence with accuracy comparable to experimentally-solved structures using known protein structure(s). It has been proven that the accuracy of a homology model depends on the target-template sequence similarity: the higher the sequence similarity, the better the model [27]. Therefore, 3MAX was employed to build a new *Pf*HDAC1 model using the SWISS-MODEL online server [28]. The obtained initial model was further refined by molecular dynamics (MD) simulations. The MD simulations’ resulting trajectory contained nineteen structures sampled every 25 ps during the simulation time. The structure with the lowest potential energy was then selected for further work.

### 2.2. Model Quality

**Figure 2:**
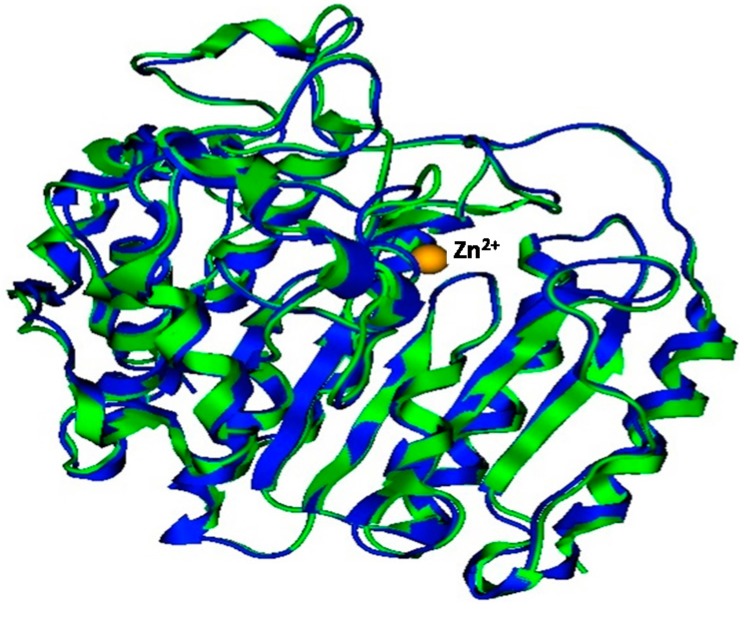
Cartoon representation of homologues superposition. Human histone deacetylase 2 (HDAC2) in blue, *Plasmodium falciparum* histone deacetylase1 (*Pf*HDAC1) in green and zinc in yellow.

The Molecular Operating Environment software package (MOE) superposition function was used to superpose the refined model and the template. The all-atom root-mean-square deviation (RMSD) between the model and the template (3MAX) was 0.85 Å, which falls within the acceptable range. All atom structure similarity can be viewed from the superposition in Figure 2.

**Figure 3:**
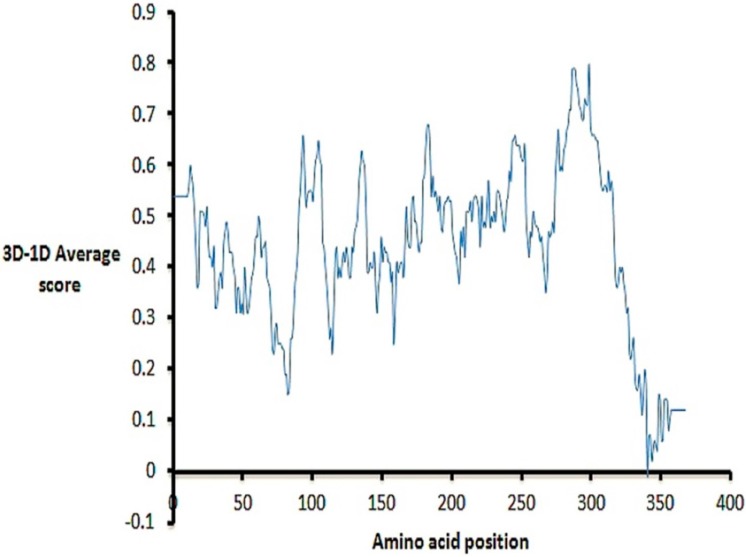
Verify3D plot of the *Plasmodium falciparum* histone deacetylase1 *Pf*HDAC1 homology model.

We further investigated the stereochemical accuracy, folding reliability and the overall quality of the model. From the Rampage server [29], 98.1% of residues were found in the favored regions of the obtained Ramachandran plot 1.4% of residues were in the allowed regions; and only two residues (0.5%) were in the plot outlier region (Figure S2). This distribution of the enzyme residues’ (ϕ,ψ) dihedral angles in the plot indicates acceptable stereochemical accuracy. Verify3D-1D differentiates between correct and incorrect protein folding depending on a compatibility score of an amino acid surrounding environment (3D) to its amino acids sequence (1D); a negative score is a sign of serious folding error [30]. From the analysis of the Verify3D result shown in Figure 3, it is clear that residues have positive scores, except for one residue, which highlights the correct model folding.

**Figure 4:**
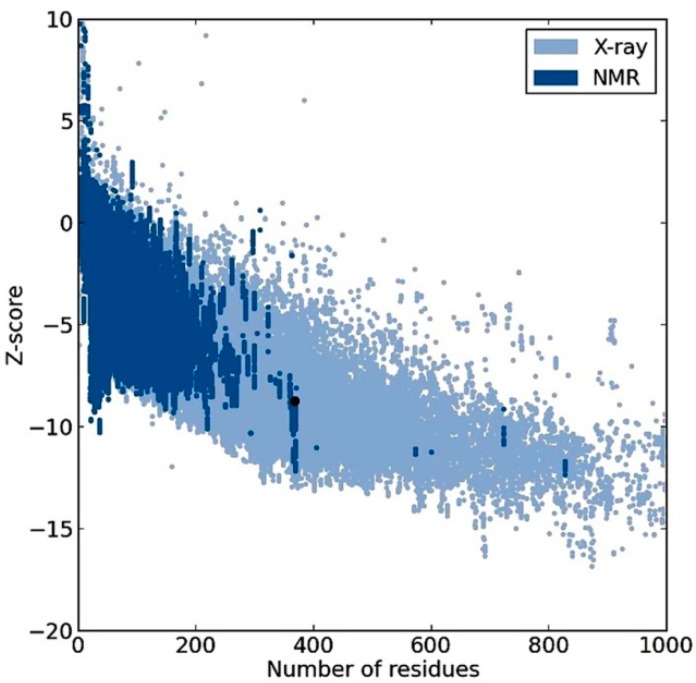
Protein Structure Assessment server (ProSA-web) result of the *Plasmodium falciparum* histone deacetylase1 *Pf*HDAC1 homology model; the black dot represents the model *z*-score.

From Figure 4, obtained from the Protein Structure Assessment (ProSA-web) server, groups of structures solved by X-ray or NMR are shown in distinctive colors, and the obtained model *z*-score was −8.76, which is placed within the range of scores typically found for native proteins of a similar size [31]. The two residues, in the outlier region in the Ramachandran plot, Ala95 and Gly178, were not matched with any residue from the template structures (*i.e*., found in the gap region in the multiple alignment shown in Figure S1), which may explain their inaccurate modeling. The same justification could explain the His336 negative score in Verify3D assessment. Interestingly, our single-template model quality assessment results were significantly higher compared to the best and most recent structure obtained from multiple templates, including our single-template 3MAX. The residues in the favored region of the Ramachandran plot were 98.1% for our model, which is significantly higher than the 91% reported for the best model obtained from multiple templates, including our single-template 3MAX [11]. Further, the number of residues in the outlier region was not reported for previous models. Moreover, our model percentage of residues scored ≥0.2 in Verify3D assessment was slightly higher (95.7%) than the best model obtained from multiple templates, including our single-template 3MAX (95%) [11]. To this level, we conclude that all results are acceptable and consistent, and the model has better assessment results than previously presented models and can be used for further work.

### 2.3. Plasmodium Falciparum Histone Deacetylase1 Model

**Figure 5:**
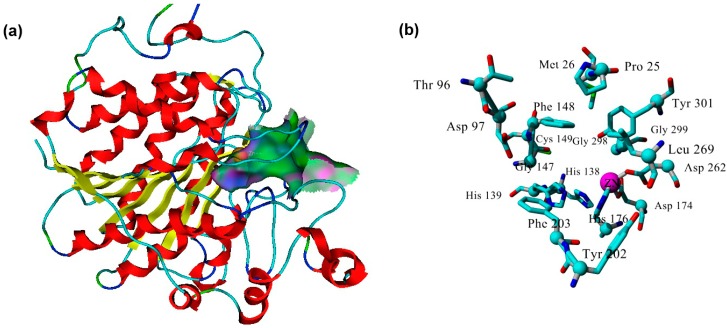
*Plasmodium falciparum* histone deacetylase1 (*Pf*HDAC1) active site. (**a**) Gaussian contour of the *Pf*HDAC1 model active site (pink represents hydrogen bonding; green represents hydrophobic contact residues; blue represents mild polar amino acids); and (**b**) key residues of the model active site.

The MD-refined model comprises a single domain following the α-/β-fold class consisting of an eight-strand β-sheet surrounded by 14 α-helices, which is similar to HDACs homologues from other species, like *Schistosoma* [32]. The enzyme active site was determined using the Alpha Site Finder embedded in MOE. The Site Finder depends on geometric methods, since no energy models are used. Instead, the relative positions and accessibility of the enzyme atoms are considered along with a rough classification of the chemical type [33]. The method is based on α-spheres, which are clustered to produce a collection of sites ranked according to the number of hydrophobic contacts made with the receptor. When the suggested sites were inspected, the site ranked first was the only one to include the enzyme catalytic Zn^2+^ that is required to accomplish the biological function of the zinc-dependent HDACs, including *Pf*HDAC1. The first ranked site is therefore selected for further work. The site contains the Zn^2+^ cofactor involved in the catalysis of the substrate occupies the active site (Figure 5a). The active site has a catalytic triad, where Zn^2+^ of the free enzyme forms a coordination bond with three amino acids, Asp174, His176 and Asp262 (Figure S3). These residues are highly conserved in Zn^2+^-dependent HDACs and correspond to Asp181, His183 and Asp269 of the template. Additional residues involved in the formation of the active site include: Pro25, Met26, Thr96, Asp97, His138, His 139, Gly147, Phe148, Cys149, Tyr202, Phe203, Leu269, Gly298, Gly299 and Tyr301 (Figure 5b).

### 2.4. Docking

#### 2.4.1. Hydroxamic Acid Inhibitor-*Pf*HDAC1 Complexes

**Figure 6:**
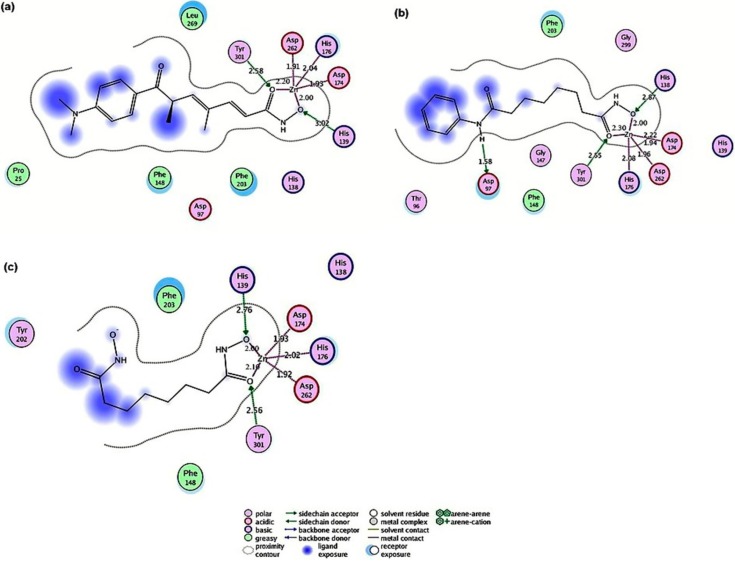
*Pf*HDAC1 model-ligand interactions. (**a**) TSA; (**b**) SAHA; and (**c**) SBHA. Bond distances are shown in angstroms.

To evaluate the docking capacity of the model, docking of known hydroxamate *Pf*HDAC1 inhibitors reported in the literature was performed [10]. Hydroxamate HDAC inhibitors are composed of a hydroxamic acid group that coordinates zinc; a hydrophobic spacer that extends through the length of the hydrophobic pocket of the HDAC enzyme; and a hydrophobic cap that seals the active site of the HDAC enzyme [8]. All inhibitors were docked in the active site of *Pf*HDAC1, and the structures showed similar ligand-enzyme docking poses presented in Figure 6. The docked inhibitors pose interactions were similar to interactions observed in crystallography-solved homologous structures (Figure S4). In all docked structures, the cofactor Zn^2+^ was pentacoordinated by Asp174, His176 and Asp262 in addition to the ligand bidentate coordination via the carbonyl and the hydroxyl group of the ligands’ hydroxamate; Figure 6. The hydroxyl group of the Tyr301 residue formed a hydrogen bond with the carbonyl oxygen of the ligand’s hydroxamate, while His139 formed a hydrogen bond with the hydroxyl of the hydroxamate moiety in the case of TSA and SBHA; Figure 6a,c. In the case of SAHA, this hydrogen bond was instead donated by His138; Figure 6b. In the enzyme-SAHA complex, Arg97 formed a hydrogen bond with the amide nitrogen of the side group (Figure 6b). This bond was not observed in other ligand enzyme complexes. Mutation studies have shown that His139, Asp174, His176, Asp262 and Tyr301 corresponding residues in human and yeast are important in the substrate catalysis process [34,35]. These residues are responsible for the stabilization of the substrate in the binding site and form part of the charge relay system necessary for the zinc-dependent hydrolysis of the acetylated lysine substrates [36]. Moreover, hydrophobic interactions involved in holding ligands within the active site include: Phe148 and Phe203 formed hydrophobic interactions with SAHA and SBHA; the two amino acids in addition to Pro25 and Leu268 formed hydrophobic interactions with TSA; Figure 6. Furthermore, the docking affinity *dG* scores of hydroxamic inhibitors ranked ligands in agreement with the experimentally obtained binding affinities represented as IC_50_ [10] (Table 1).

**Table 1:** Binding energy calculated from MD-simulated *Pf*HDAC1 complexes (kcal·mol^−1^).

Ligand	IC_50_ (µM) ^a^		Score	Binding Energy (kcal·mol^−1^)
	CQ ^b^ Resistant	CQ ^b^ Sensitive		
**SAHA**	1.78	0.94	−5.15	280.81
**SBHA**	0.8	1.3	−6.52	281.92
**TSA**	0.008	0.11	−6.92	308.54
**Valproic acid**	N.A. ^c^	N.A. ^c^	−5.13	219.67

^a^ Antiproliferative potencies against *P. falciparum in vitro* obtained from Andrews *et al.* [10]; ^b^ CQ = chloroquine; ^c^ N.A. = Not available. SAHA: suberoylanilide hydroxamic acid; SBHA: suberoyl bis-hydroxamic acid; TSA: trichostatin A; and CQ: chloroquine.

#### 2.4.2. Valproic Acid-*Pf*HDAC1 Complex

**Figure 7:**
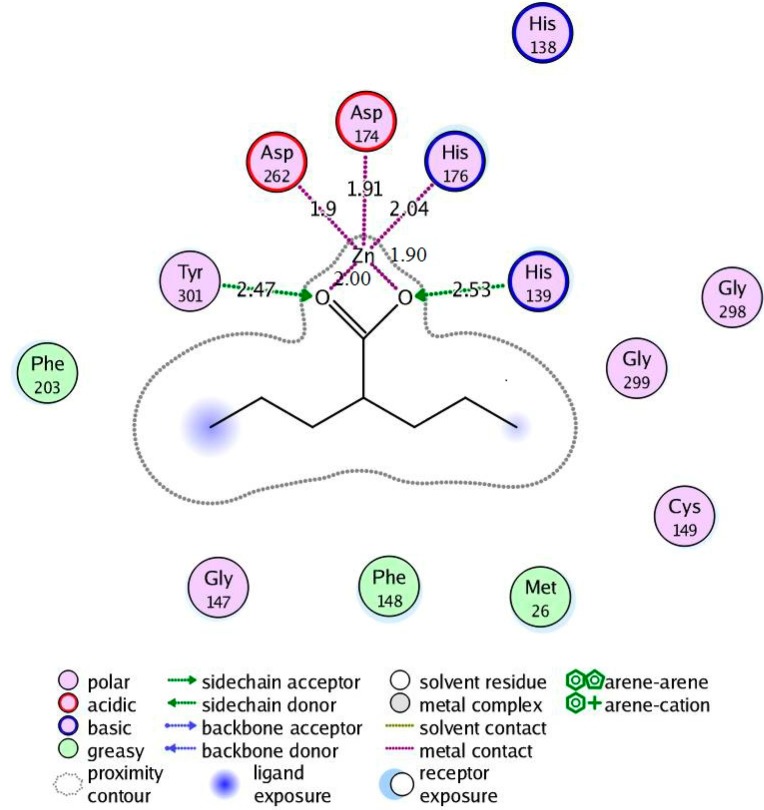
Valproic acid-*Pf*HDAC1 complex interactions.

Regarding the valproic acid-enzyme complex, valproic acid showed bidentate coordination with the enzyme Zn^2+^ via the carboxyl carbonyl oxygen and hydroxyl oxygen. Tyr301 and His139 donated hydrogen bonds to the acid carbonyl and hydroxyl oxygen, respectively, which is similar to the coordination fashion observed in the hydroxamic acid derivative enzyme complexes. Enzyme hydrophobic interactions with the molecule alkyl part involved Met26, Phe148 and Phe203, which were also similar to corresponding interactions with the hydroxamate-enzyme complexes (Figure 7).

### 2.5. Molecular Dynamics Simulations

Molecular docking has been successful in binding pose prediction, but it has also failed in expecting binding affinity on many occasions [37,38]. In order to relax the geometries, to get an insight into the stability of the ligand-enzyme complexes and to obtain more reliable binding energies, 5-ns MD simulations were performed on each ligand enzyme complex followed by rigorous MD simulation trajectory analysis. Apart from the other MD simulation objectives mentioned above in this paragraph, from our previous experience in molecular modeling, which agrees with Moonsamy *et al*. [39], a ligand in even the best docking pose may move away from the binding site within a few picoseconds when subjected to MD simulations. Therefore, we are convinced that any docking calculations not followed by MD simulations at least for hundreds of picoseconds are less reliable.

**Figure 8:**
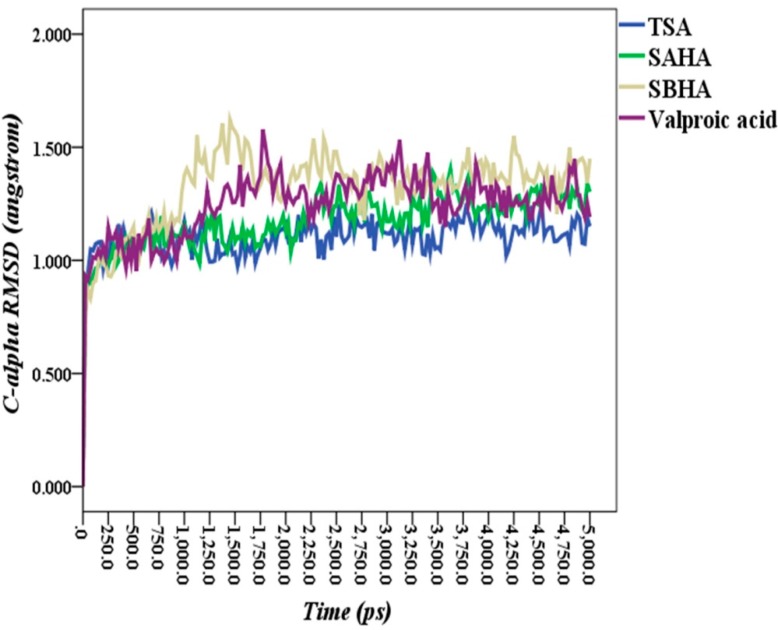
Root-mean-square deviation (RMSD) of Cα atoms of enzyme-ligand complexes *versus* time.

The Cα-RMSD of each enzyme ligand complex MD simulation trajectory *versus* time is shown in Figure 8. The average RMSDs of Cα positions along simulation trajectories *versus* the time of each structure trajectory were 1.10, 1.17, 1.32 and 1.24 Å for TSA, SAHA, SBHA and valproic acid enzyme complexes, respectively.

**Figure 9:**
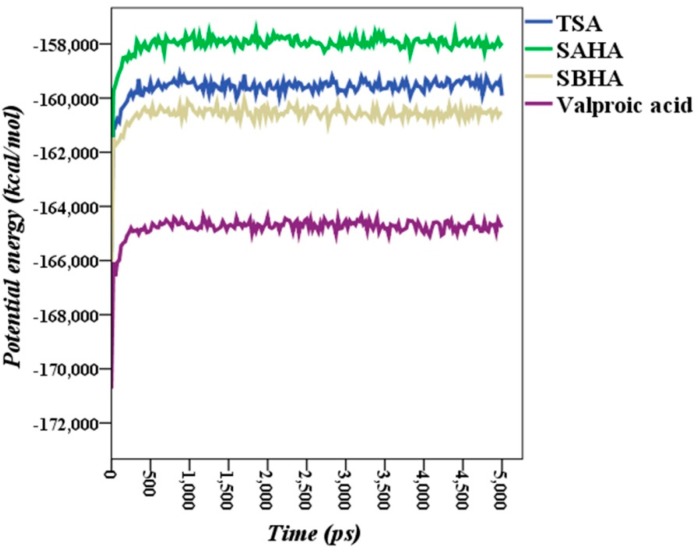
Potential energies (kcal·mol^−1^) of *Plasmodium falciparum* histone deacetylase1 ligand complexes during molecular dynamics simulation.

The potential energy of each enzyme-ligand complex along the 5-ns simulation period showed that the complexes equilibrated within a maximum of about 500 ps. Once the equilibration time point was passed, the potential energy had plateaued, and the variability as below 800 kcal·mol^−1^ along the remaining 4500 ps simulation time, as shown in Figure 9. The RMSD values together with the MD steady potential energies during simulations imply that the enzyme ligand complex systems have good stability and reliability.

### 2.6. Theoretical Binding Energies

The calculated theoretical binding energies of ligands obtained in this work and their corresponding IC_50_ from *P. falciparum in vitro* growth inhibition assays obtained from the literature [10] are presented in Table 1. The calculated binding energies are in good agreement with the ligands’ IC_50_ values. No corresponding experimental data are available for comparison with valproic acid. The calculated binding energy of valproic acid (219.67 kcal·mol^−1^) was around 72% of the highest calculated TSA binding energy value and 79% of the approved anticancer SAHA (Table 1).

## 3. Materials and Methods

### 3.1. Homology Modeling

The FASTA format of the 449 amino acid sequence of *Pf*HDAC1 was retrieved from the UniprotKB database (Accession Number Q7K6A1) [21]. BLAST searching was performed to obtain the template with the highest sequence identity to *Pf*HDAC1 [23]. Human HDAC-2 (PDB: 3MAX) was found to have the highest sequence identity with *Pf*HDAC1 [24]. The sequence of *Pf*HDAC1 together with 3MAX was submitted to the SWISS-MODEL server for homology model building [28].

### 3.2. Model Refinement

The SWISS-MODEL-generated model was further refined by MD simulations using the YASARA program [40], employing the molecular dynamics macro (md_refine). In brief, the model was subjected to MD simulations using the YASARA2 force-field for 500 ps at 298° K using the NVT canonical ensemble. The default simulation parameters defined by the macro were used during the simulations. The detailed MD refinement procedure was described elsewhere [41].

### 3.3. Model Quality Validation

The quality of the model was carefully examined using different bioinformatics online tools. The Ramachandran (ϕ,ψ) dihedral angle plot generated by the RAMPAGE server [29] was used to evaluate the model stereochemical quality. The Verify3D server [30] was employed to check the model folding reliability. ProSA-web (*z*-score) was used to check the overall quality of the 3D model [31].

### 3.4. Ligands Preparation

MOE software was used for ligand preparation [42]. All ligand structures were obtained from the PubChem database: TSA (CID 444732), suberoylanilide hydroxamic acid (SAHA) (CID 5311), suberoyl bis-hydroxamic acid (SBHA) (CID 5173) and valproic acid (CID 3121). Ligand structures are shown in Figure 1. The protonation state of hydroxamic acid complexes with Zn^2+^ in the active site of zinc-dependent HDACs is controversial. Experimentally, it is difficult to determine the position of the proton directly. However computational studies suggest negative hydroxamate in the active site [43,44], while another study suggests a neutral hydroxamic acid inhibitor [45]. A recent study’s results have strongly suggested negative hydroxamate-Zn^2+^ coordination and explained several experimental observations [46]. Therefore, hydroxamic acid inhibitors and valproic acid 3D structures were imported together in a single MOE database, and thereafter, their hydroxyl groups were deprotonated. The generated hydroxamates and valproate were energy minimized to within an rms gradient of 0.1 kcal·mol^−1^·Å^−1^ using the MMFF94x force-field [47].

### 3.5. Docking

All molecular docking of ligands into *Pf*HDAC1 was performed using MOE [42]. Potential binding sites in the homology model were recognized using the Alpha Site Finder [48]. Once the active site was identified, ligands were docked into the selected site using the triangle matcher placement method [42]. Thirty poses were retained for each ligand and scored using the London ΔG function [42]. The retained poses were further refined by energy minimization to 0.1 rms·kcal·mol^−1^·Å^−1^ using the CHARMM27 molecular mechanics force field [49] and rescored using the affinity ΔG scoring function [42]. In the docking, the flexible-ligand rigid-protein approach was employed, where flexible ligand conformations were generated using the Monte Carlo algorithm. The pose with the best refining score of each ligand was selected for further work.

### 3.6. Molecular Dynamics Simulation

Independent molecular dynamics simulations of all ligand-enzyme complex structures were performed using the YASARA program [40]. All structures’ geometry was minimized to within an rms gradient of 0.1 kcal·mol^−1^·Å^−1^ using the AMBER99 force-field [50]. All systems were independently contained in a simulation cell of 79.98 × 79.98 × 79.98 Å surrounded by periodic boundary conditions and solvated with water TIP3P molecules [51], and thereafter, simulated annealing minimization of the solvent was performed. Residue protonation states were assigned in relation to calculated p*K*_a_ values and physiologic simulation, pH 7.4 [52]. Sodium and chloride ions were randomly added to the solvated structures to neutralize the cell and achieve a 0.9% NaCl ion mass fraction (physiological condition). To relax the structures geometry, 5-ns MD simulations were performed and followed by a final energy minimization step. MD simulations were performed at 298 K using the NVT canonical ensemble. The simulations were performed in multiple time steps of 1.25 fs for intramolecular forces and 2.5 fs for intermolecular forces. The particle mesh Ewald method at a cutoff of 7.86 Å was used for long range electrostatic force calculations [53]. MD simulations were sampled every 25 ps, resulting in 200 snapshot trajectories.

### 3.7. Ligand-Enzyme Complex Theoretical Binding Energy Calculation

In YASARA, theoretical binding energy is obtained by calculating the unbound ligand energy at infinite distance and subtracting the energy of the bound state. A larger positive binding energy is defined in this context as a more favorable binding for a given force-field [40,54]. All theoretical binding energies were calculated after MD simulations and final energy minimization.

## 4. Conclusions

A homology model of *Pf*HDAC1 based on the crystal structure of human HDAC2 is presented. The model was reliable in terms of stereochemical accuracy, folding reliability and overall correctness assessed by online bioinformatics tools. The model quality was better than previously presented models. The model has shown similar binding site residues observed in zinc-dependent HDAC from other species. Docking affinity *dG* scores agreement with the corresponding experimental IC_50_ obtained from the literature implies that the model and the docking methodology are dependable and can be used to screen virtual compound libraries for *Pf*HDAC1 inhibitors. In this work, MD simulations supported the reliability and stability of the docking poses and reinforced the theoretical binding energy calculations. The valproic acid calculated theoretical binding energy suggests that it is rational to extend studying this drug *in vitro* and in malaria mouse model as a potential *Pf*HDAC1 inhibitor, particularly when taking into account the high maximum dose of the drug. Currently, we are investigating the effect of valproic acid on *P. falciparum* growth *in vitro* and in mice infected with *P. berghei*, which causes rodent malaria.

## Supplementary Materials

Supplementary materials can be found at http://www.mdpi.com/1422-0067/16/02/3915/s1.
